# The class A repeats of LRP5 are required for normal development of bone, retinal vasculature and mammary gland *in vivo*

**DOI:** 10.1242/dmm.052280

**Published:** 2025-11-11

**Authors:** Cassandra R. Diegel, Megan N. Michalski, John L. Ubels, Gabrielle Foxa Wiartalla, Cheng-Mao Lin, Zhendong A. Zhong, Mitchell J. McDonald, Nicole J. Ethen, Madison Brookshire, Zachary B. Madaj, Mingxuan Xia, Paul R. Gavine, David A. Antonetti, Bart O. Williams

**Affiliations:** ^1^ Department of Cell Biology, Van Andel Institute, 333 Bostwick Ave., NE, Grand Rapids, MI 49503, USA; ^2^ Kellogg Eye Center, Department of Ophthalmology and Visual Sciences, University of Michigan School of Medicine, 1000 Wall St, Ann Arbor, MI 48105, USA; ^3^ Bioinformatics and Biostatistics Core, Van Andel Institute, 333 Bostwick Ave., NE, Grand Rapids, MI 49503, USA; ^4^ Oncology Translational Research Asia, Johnson & Johnson Innovative Medicines, Shanghai 200233, China; ^5^ Oncology Discovery, Johnson & Johnson Innovative Medicines, Beerse 2340, Belgium

**Keywords:** LRP5, Wnt, LDLR type A repeats, Bone, Retina

## Abstract

Low-density lipoprotein-related receptor 5 (LRP5) is an LDLR family member with well-defined roles in mediating Wnt signaling. Its domain structure includes four LDLR class B and three LDLR class A repeats. Class B repeats mediate binding with Wnt ligands and other effectors, while the role of the LRP5 class A repeats, known to interact with apolipoproteins within the LDLR, is unclear. Complete loss of the *LRP5* gene in humans causes osteoporosis pseudoglioma, a syndrome characterized by early-onset osteoporosis and changes in retinal vascularization. We and others have previously created mice and rats completely deficient in LRP5 and reported the presence of bone and retinal vascularization defects. In this study, we created an allele of *Lrp5* in mice in which the entire protein except for the class A repeats is present and expressed from the endogenous locus. Unlike *in vitro* studies using ectopic overexpression of LRP5, our *in vivo* data demonstrate that the class A repeats are essential for several normal LRP5 functions, including bone homeostasis, retinal vascularization and mammary gland development – phenotypes similar to those observed in *Lrp5* null mice.

## Teaser

**Summary:** The LDLR class A repeats of LRP5 are essential *in vivo* for bone integrity, retinal vascularization and mammary development, underscoring their critical role in normal LRP5-mediated physiological functions.

## INTRODUCTION

Low-density lipoprotein-related receptor 5 (LRP5) was first identified based on its homology to the low-density lipoprotein receptor (LDLR) (Dong et al., 1998; Hey et al., 1998; Kim et al., 1998). It contains the class A and B repeats present in all LDLR family members (Yang and Williams, 2017). Class B repeats have a B-propeller structure surrounded by three EFG-like repeats with a conserved YWTD motif, critical for ligand release and receptor recycling following internalization into endosomes (Jeon and Blacklow, 2003; Beglova and Blacklow, 2005). In LRP5, the four type B repeats mediate binding to Wnt ligands (Joiner et al., 2013). Similar to the highly homologous LRP6 protein (Joiner et al., 2013), LRP5 is a requisite co-receptor with members of the Frizzled family of seven-transmembrane proteins to allow Wnt ligands to activate canonical (β-catenin-dependent) signaling and activate target gene transcription (Joiner et al., 2013). Other ligands for the class B repeats of LRP5 include Dickkopf-1 (DKK1) and sclerostin (SOST), which directly bind LRP5 and block Wnts from binding to activate the canonical pathway (Mason and Williams, 2010).

The role of the class A repeats in LRP5 function is less clear. Class A repeats are ∼40 amino acids, with three internal disulfide bonds coordinating a calcium ion. In addition, they contain a C-terminal cluster of conserved acidic residues (Beglova and Blacklow, 2005; Pedersen et al., 2014). In the LDLR, seven class A repeats are present at the amino terminus and mediate the binding of low-density lipoproteins (Esser et al., 1988). In LRP5, three class A repeats are found between four N-terminal class B repeats and the transmembrane domain. The requirement for these class A repeats within LRP6 was examined following transient transfection of various expression plasmids into HEK293T cells. LRP6 variants lacking the class A repeats activate β-catenin-dependent signaling to a similar level as wild-type (WT) LRP6 when either is ectopically overexpressed in HEK293T cells (Brennan et al., 2004). However, to our knowledge, the requirement for class A repeats in LRP6 under conditions of physiological expression levels has not been evaluated, and their role in LRP5 has not been reported for any system.

Our understanding of LRP5 functions comes primarily from evaluating phenotypes of human patients and the analogous genetically engineered mouse models lacking LRP5 (Maupin et al., 2013). Humans who are homozygous for inactivating mutations in LRP5 develop osteoporosis pseudoglioma (OPPG), a syndrome characterized by bone and eye abnormalities (Gong et al., 2001). OPPG patients suffer from recurrent bone fractures due to severe osteoporosis, which manifests in early childhood. Patients are often blind because of defective vasculature in the retina and retrolental membranes. We and others have shown that mice (Kato et al., 2002; Holmen et al., 2004; Clément-Lacroix et al., 2005; Cong and Zhang, 2015) or rats (Ubels et al., 2022, 2020) lacking LRP5 develop skeletal and retinal defects similar to those seen in human OPPG patients. Human patients with loss-of-function variants in *LRP5* can also develop familial exudative vitreoretinopathy (FEVR), a disease characterized by a paucity of blood vessels at the retinal periphery and hemorrhages of other regional blood vessels (Le et al., 2023). Our previously reported LRP5 knockout rats appear to be a good model of FEVR (Cong and Zhang, 2015; Ubels et al., 2022).

This work defines the requirement for the class A LRP5 repeats under physiological conditions in normal development. To do this, we created mice with an *Lrp5* allele that specifically and uniquely lacks the class A repeats but retains all other parts of the LRP5 protein (*Lrp5*^ΔLDLRA^). Because the sequences encoding the three class A repeats are contained entirely and uniquely within exons 18 and 19 of the mouse *Lrp5* gene, we used CRISPR/Cas9-mediated genetic editing to delete these two exons. The resulting gene ­maintained normal levels of LRP5 protein expression despite lacking these repeats. We found that mice homozygous for the *Lrp5*^ΔLDLRA^ allele had reduced bone mass and retinal vascularization phenotypes reminiscent of those seen in mice homozygous for null alleles of *Lrp5*. Thus, this work is the first to demonstrate that class A repeats are necessary for the normal functions of LRP5.

## RESULTS

**Fig. 1.**
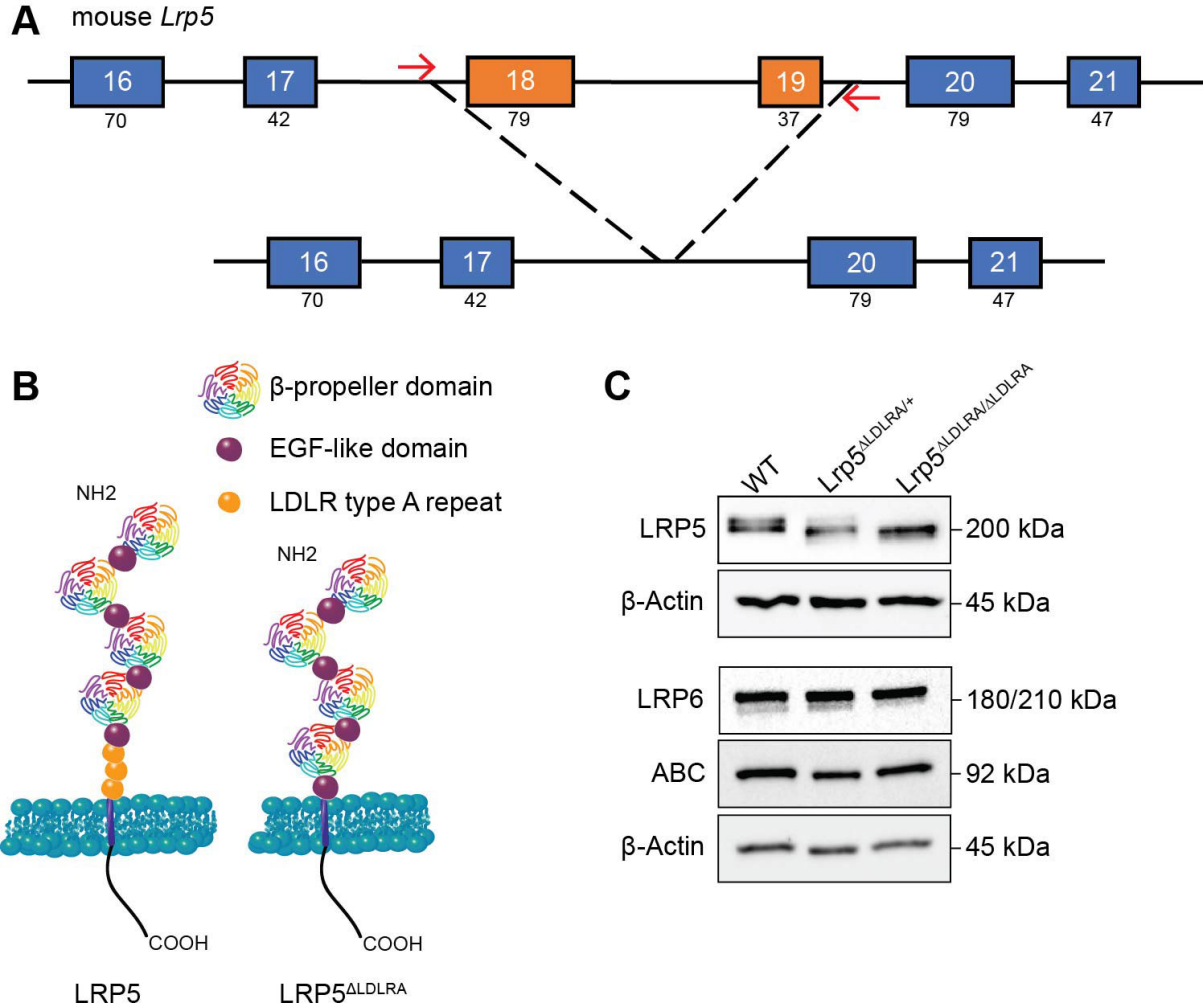
***Lrp5*^ΔLDLR^ animal model schematic and LRP5 protein expression in mouse embryonic fibroblasts (MEFs).** (A) CRISPR/Cas9 targeting schematic of intron 17 and 19 of mouse *Lrp5*. The numbers in the boxes depict the exon number, with the number of amino acids listed below. Red arrows indicate the general sgRNA location. (B) Model of wild-type LRP5 and LRP5^ΔLDLR^ protein on the cell surface. (C) *Lrp5* wild-type (WT), *Lrp5^ΔLDLR/+^* and *Lrp5*^ΔLDLRA/ΔLDLRA^ MEFs were collected to immunoblot for LRP5, LRP6 and active β-catenin (ABC). β-actin was used as loading control.

To define the function of the LRP5 LDLR type A repeats for LRP5, we generated a new *Lrp5*^ΔLDLRA^ mouse model using CRISPR/Cas9 gene editing. Simultaneous zygotic injection of Cas9 mRNA and guide RNAs recognizing sequences within introns 17 and 19 was used to excise exons 18 and 19 (Fig. 1A) of the *Lrp5* gene. These exons uniquely and specifically encode the LDLR type A region (Fig. 1B). Founders were identified by PCR genotyping followed by Sanger sequencing validation of the amplified PCR products. We analyzed two different founders to confirm that any phenotypes were likely the result of the intended modification and not a byproduct of the gene modification process. Both founders (*Lrp5*^em4Vari^ and *Lrp5*^em5Vari^) had exons 18 and 19 excised, but the indel was slightly different which is typical for CRISPR-Cas9 edited models (Ubels et al., 2022). Data associated with founder *Lrp5*^em4Vari^ are detailed in the paper; data related to *Lrp5*^em5Vari^ are presented in Fig. S1 and Table S1.

Tissue was collected from mice WT (‘+’), heterozygous and homozygous for the *Lrp5*^ΔLDLRA^ allele to assay RNA and protein changes. Quantitative real-time PCR (qRT-PCR) showed the absence of exons 18 and 19 from mRNA in homozygous *Lrp5*^ΔLDLRA^ mice (Fig. S1). LRP5 protein was expressed in heterozygous and homozygous *Lrp5*^ΔLDLRA^ tissue with approximately a 12 kDa smaller product, as predicted from the loss of 116 amino acids (Fig. 1C). LRP6 expression was not affected by the loss of type A repeats in LRP5 based on western blot analysis (Fig. 1C).

### Evaluating genetic interactions between the *Lrp5*^ΔLDLRA^ allele and a null allele for *Lrp6*

LRP6-deficient mice die shortly after birth, while embryos homozygous for null mutations in both *Lrp5* and *Lrp6* (*Lrp5*^KO/KO^; *Lrp6*^KO/KO^) die soon after gastrulation, becoming visibly smaller with severe developmental delays by embryonic day (E)7.5, and fail to form mesoderm (Kelly et al., 2004). *Lrp5*^ΔLDLRA/ΔLDLRA^; *Lrp6*^KO/KO^ embryos are also embryonic lethal (Fig. S2A).

We previously showed that mice homozygous for the null allele of *Lrp5* and heterozygous for a null (KO) allele of *Lrp6* (*Lrp5*^KO/KO^; *Lrp6*^KO/+^) display highly penetrant limb patterning defects ranging from synostoses to the absence of an entire limb (Holmen et al., 2004). To compare *Lrp5*^ΔLDLRA^ to the null allele in this context, we created *Lrp5*^ΔLDLRA/ΔLDLRA^; *Lrp6*^KO/+^ mice. We found that limb patterning in *Lrp5*^ΔLDLRA/ΔLDLRA^; *Lrp6*^KO/+^ mice was normal at E18.5 (*n*=6) and at 3 months (*n*=13) (Fig. S2B). Thus, the class A repeats of LRP5 are dispensable for function in normal limb patterning.

### *Lrp5*^ΔLDLRA^ mice have smaller, thinner bones than those of littermate controls

**Fig. 2.**
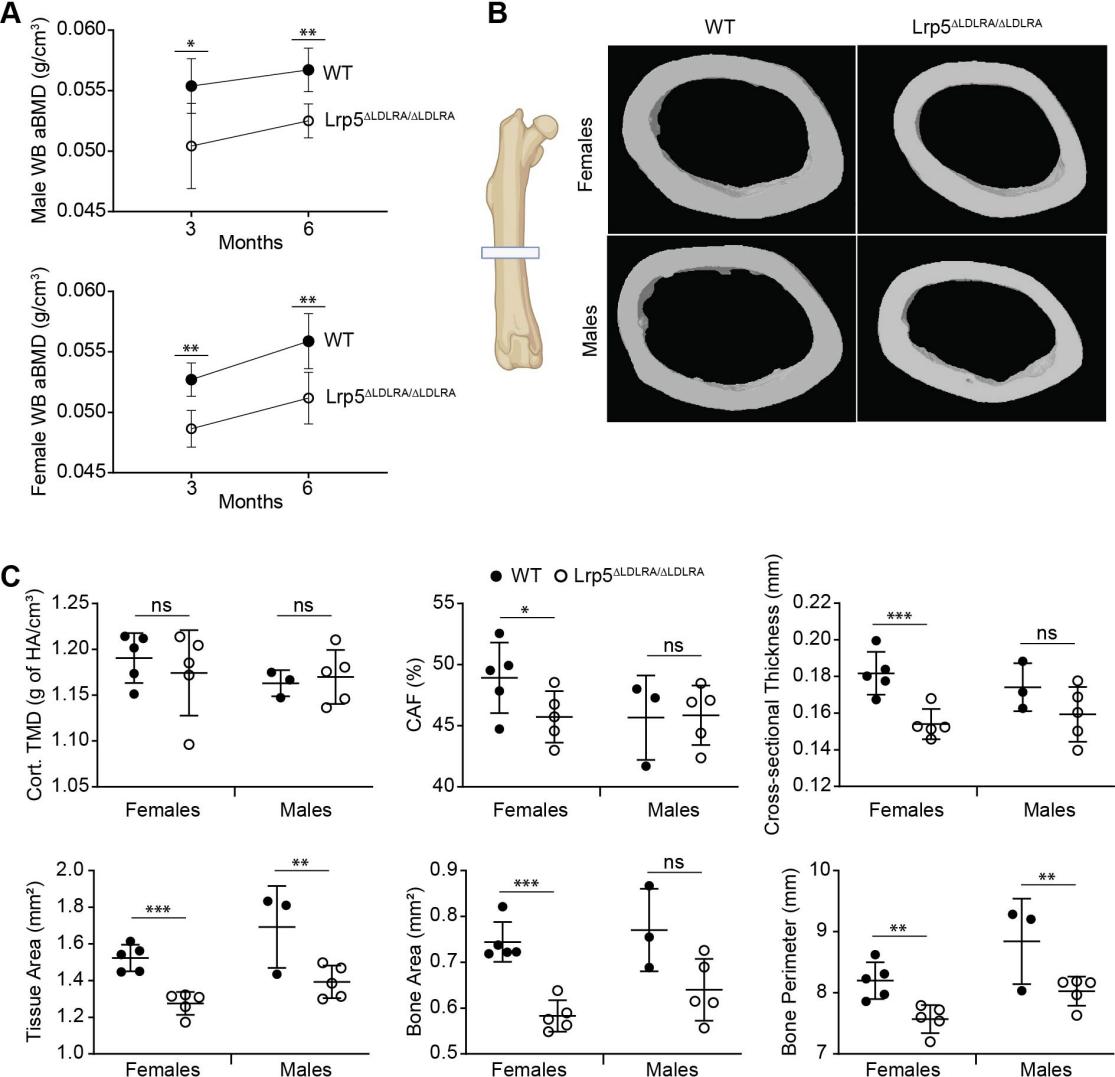
**Cortical bone analysis of *Lrp5*^ΔLDLR/ΔLDLR^ mice via micro-computed tomography (µCT).** (A) Longitudinal whole-body (WB) areal bone mineral density (aBMD) measured by dual-energy X-ray absorptiometry (DXA) of 3- and 6-month-old *Lrp5*^ΔLDLRA/ΔLDLRA^ mice and wild-type (WT) littermates. For DXA graphs, averages of each group are represented; upper and lower lines represent s.d. Number of animals analyzed: 3 months, five mice per sex per genotype; 6 months, five females per sex per genotype and five male WT and four *Lrp5*^ΔLDLRA/ΔLDLRA^ mice. (B) Representative µCT images of cortical bone in the midshaft of WT and *Lrp5*^ΔLDLRA/ΔLDLRA^ femurs at 3 months of age. Femur image created in BioRender by Diegel, C. (2025). https://BioRender.com/0o0kdfh. This figure was sublicensed under CC-BY 4.0 terms. (C) Cortical bone indices of *Lrp5*^ΔLDLRA/ΔLDLRA^ mice and WT controls were measured. These parameters included tissue mineral density (TMD), cortical area fraction (CAF), cross-sectional thickness, tissue area, bone area and bone perimeter. For all µCT graphs, means of each group are indicated by the middle line; upper and lower lines represent s.d. ns, not significant; *P<0.05, **P<0.01, ***P<0.001 (robust linear regression). Number of animals analyzed: five female mice per genotype and three male WT and five male *Lrp5*^ΔLDLRA/ΔLDLRA^ mice.

*Lrp5* knockout mice recapitulate the bone phenotypes seen in human OPPG patients (Kato et al., 2002; Holmen et al., 2004; Clément-Lacroix et al., 2005; Cui et al., 2011; Sawakami et al., 2006). They a have significantly lower bone mineral density (BMD), as determined by dual-energy X-ray absorptiometry (DXA), and decreased cancellous bone characteristics as determined by high-resolution micro-computed tomography (µCT). We performed bone assessments on mice homozygous for the *Lrp5*^ΔLDLRA^ allele. Whole-body areal BMD (aBMD) by DXA (Fig. 2A) for female and male homozygous animals at 3 and 6 months of age was statistically lower than that for sex- and age-matched WT littermates. *Ex vivo* µCT analysis indicated no significant reductions in femoral trabecular bone characteristics, but changes in cortical bone morphometric indices occurred. Representative cortical bone µCT images showed a slight decrease in bone size and thickness (Fig. 2B). The cortical tissue mineral density was not significantly different between genotypes and sexes. In general, *Lrp5*^ΔLDLRA^ homozygous females had a stronger cortical phenotype than that of males, with a 7% decrease in cortical area fraction (CAF), a 15% decrease in cortical cross-sectional thickness, a 16% decrease in tissue area, a 22% decrease in bone area and an 8% decrease in bone perimeter (Fig. 2C). These indices indicated that *Lrp5*^ΔLDLRA^ female mice have smaller, thinner bones at 3 months of age than those of littermate controls. *Lrp5*^ΔLDLRA^ males, on the other hand, had less severe cortical changes, except for an 18% decrease in tissue area and a 9% decrease in bone perimeter (Fig. 2C). In contrast, our second founder, *Lrp5*^em5Vari^, showed statistically significant cortical changes in both sexes (Table S1).

### *Lrp5*^ΔLDLRA/ΔLDLRA^ mice have retinal vascularization defects

**Fig. 3.**
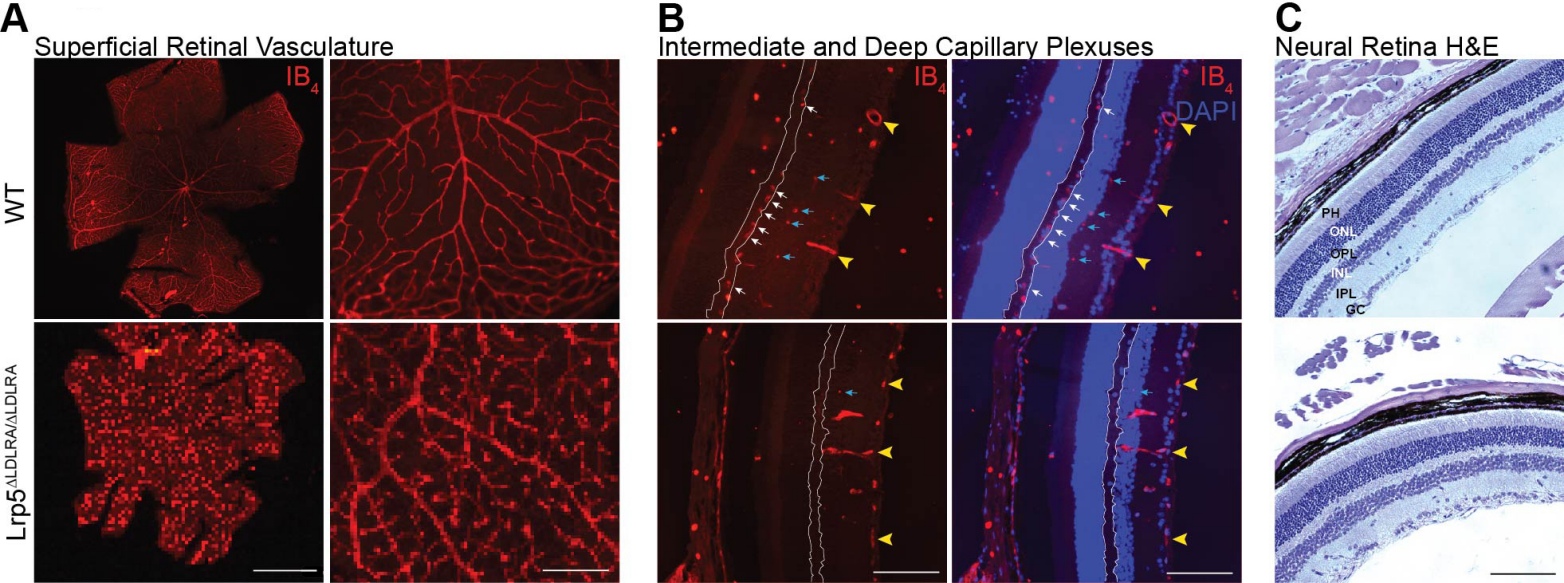
**Retinal vasculature in 3-month-old *Lrp5*^ΔLDLRA/ΔLDLRA^ mice.** (A) Superficial vasculature was analyzed using flat-mounted retinas of WT and *Lrp5*^ΔLDLRA/ΔLDLRA^ animals stained with Alexa Fluor 594-conjugated isolectin B_4_ (IB_4_; *N*=8 per genotype)_._ *Lrp5*^ΔLDLRA/ΔLDLRA^ retinas have dense retinal vasculature that does not branch normally, with vessels ending in terminal bulbs. Scale bars: 1 mm (left), 0.2 mm (right). (B) Frozen sections stained with IB_4_ (left images) and DAPI (merged images on right). WT retinas have a normal deep vascular plexus (white arrows) in the outer plexiform layer (outlined in white) and intermediate vascular plexus (blue arrows) in the inner plexiform layer. *Lrp5*^ΔLDLRA/ΔLDLRA^ retinas do not have a deep plexus, and the intermediate plexus is sparse. Yellow arrowheads indicate superficial retinal vessels present in both WT and knockout retinas. In knockout retinas, some superficial vessels extend into the outer nuclear layer as seen in the merged image. Retinal layers are labeled in C (*n*=5 per genotype). Scale bars: 200 mm. (C) Hematoxylin and Eosin (H&E) staining of paraffin sections shows that the neural retina appears normal in both genotypes. PH, photoreceptors; ONL, outer nuclear layer; OPL, outer plexiform layer; INL, inner nuclear layer; IPL, inner plexiform layer; GC, ganglion cells (*n*=2 WT and 3 *Lrp5*^ΔLDLRA/ΔLDLRA^). Scale bar: 200 mm.

We also investigated whether mice homozygous for the *Lrp5*^ΔLDLRA^ allele have retinal vascularization phenotypes like those homozygous for null alleles of *Lrp5* (Holmen et al., 2004; Chen et al., 2012; Huang et al., 2016; Wang et al., 2016). We found that *Lrp5*^ΔLDLRA/ΔLDLRA^ mice had a relatively dense superficial retinal vasculature with vessels that end in terminal bulbs (Fig. 3A) rather than a normal branching pattern. This is evidence of neovascularization in response to oxygen deficiency early in development. The vasculature is like that seen in the retinas of mice homozygous for a null allele of *Lrp5* (Chen et al., 2012; Huang et al., 2016; Wang et al., 2016; Xia et al., 2010). The dense vasculature is in marked contrast to the sparse superficial vasculature of *Lrp5* null rats (Ubels et al., 2022, 2020). As in other *Lrp5* knockout mouse models, and in contrast to *Lrp5* null rats, there were no auto-fluorescent exudates in *Lrp5*^ΔLDLRA/ΔLDLRA^ mice. The deep vascular plexus was absent in *Lrp5*^ΔLDLRA/ΔLDLRA^ mice (Fig. 3B), as in *Lrp5* null mice and rats (Ubels et al., 2022, 2020; Wang et al., 2016). Few intermediate vessels were present, and some superficial vessels extended into the inner nuclear layer, apparently compensating for the lack of deep and intermediate vessels.

The layering and morphology of the neural retina in *Lrp5*^ΔLDLRA/ΔLDLRA^ mice appeared normal. The layers were well organized, and photoreceptor outer segments were clearly visible (Fig. 3C), consistent with previous reports of mice homozygous for the null allele of *Lrp5* (*Lrp5*^KO/KO^) (Xia et al., 2008). In contrast, the outer nuclear layer was disorganized in *Lrp5*^KO/KO^ rats and the electroretinogram was attenuated. The presence of photoreceptors in *Lrp5*^ΔLDLRA/ΔLDLRA^ mice was in marked contrast to the highly disorganized retinas of *Lrp5* null rats, which have no photoreceptors (Ubels et al., 2022). Although the vasculature of *Lrp5*^ΔLDLRA/ΔLDLRA^ mice is quite abnormal, it can apparently supply adequate oxygen and nutrients to support development and maintenance of the neural retina. As shown below, however, the abnormal vasculature of *Lrp5*^ΔLDLRA/ΔLDLRA^ mice does not support a normal response to light.

### Scotopic electroretinography analysis of *Lrp5*^ΔLDLRA/ΔLDLRA^ mice

**Fig. 4.**
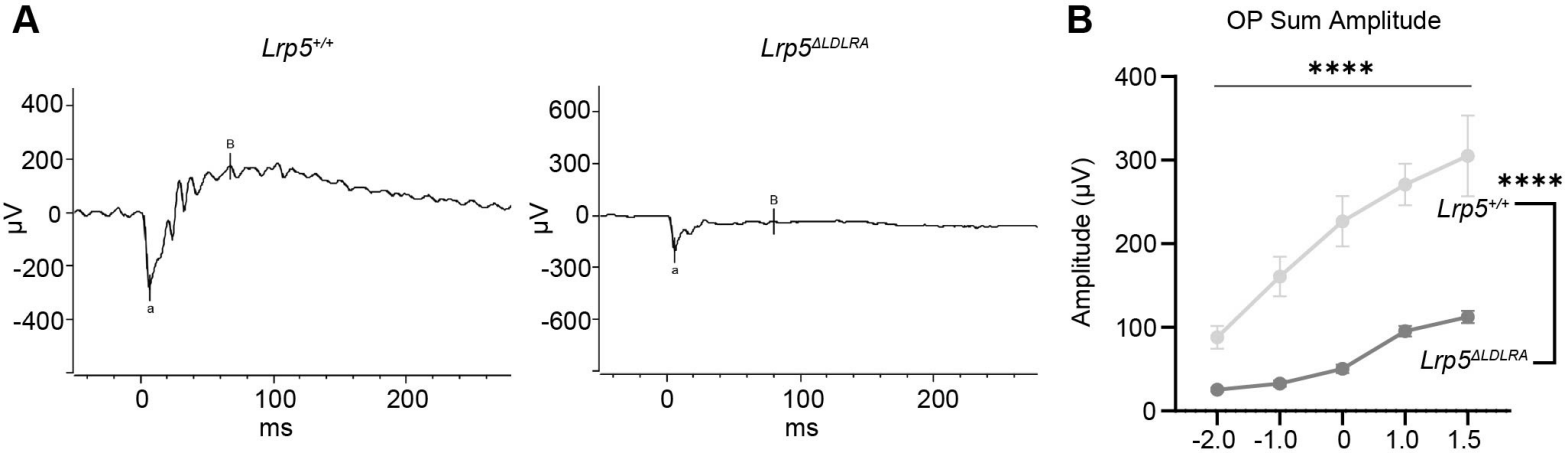
**Retinal function is compromised in *Lrp5*^ΔLDLRA/ΔLDLRA^ mice.** (A) Electroretinograms under scotopic conditions revealed reduced retinal function in *Lrp5*^ΔLDLRA/ΔLDLRA^ knockout mice with attenuation of a- and b-waves. (B) Quantitative analysis of scotopic b-wave oscillatory potential (OP) amplitudes measured at selected flash intensities (−1.0, 0.0 and +1.0 log cd.s/m²) in WT and *Lrp5*^ΔLDLRA/ΔLDLRA^ knockout mice. Knockout animals exhibited a significant reduction in OP amplitudes across all tested intensities, indicating impaired rod-driven responses in bipolar and amacine cells. Data are presented as mean±s.e.m. ****P≤0.0001 (two-way ANOVA).

To determine whether altered vascularity impacts retinal function, we performed scotopic electroretinography (ERG) to measure a- and b-waves. The b-wave oscillatory potentials (OPs), which reflect inner retinal activity, particularly from amacrine and bipolar cells, were also analyzed. ERG a- and b-amplitudes were markedly attenuated in *Lrp5*^ΔLDLRA/ΔLDLRA^ mice (Fig. 4A). Across all stimulus intensities tested, *Lrp5*^ΔLDLRA/ΔLDLRA^ mice exhibited significantly reduced summed OP amplitudes compared to those of WT animals (*P*<0.0001) (Fig. 4B; Fig. S3). These results indicated that altered retinal vascularity due to loss of class A repeats of LRP5 leads to profound deficits in photoreceptor function, as indicated by reduced a-wave amplitude, and inner retinal activity, as indicated by reduced OP amplitude. These ERG changes occur despite histologically normal retinal cellular structure. We conclude that the absence of the three type A domains of the LRP5 Wnt co-receptor has a similar effect on the development of the retinal vasculature and retinal function in mice as complete absence of LRP5 (Chen et al., 2012; Huang et al., 2016; Wang et al., 2016; Xia et al., 2010).

### *Lrp5*^ΔLDLRA/ΔLDLRA^ mice have altered mammary development

**Fig. 5.**
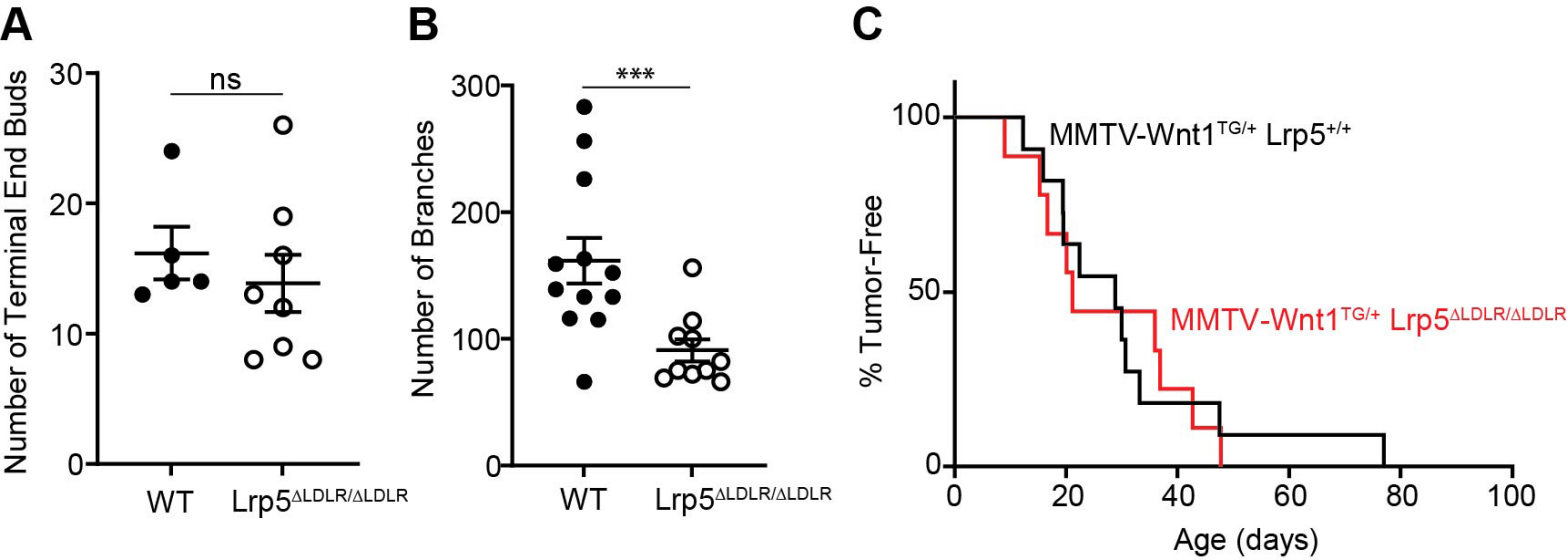
***Lrp5*^ΔLDLRA/ΔLDLRA^ mammary glands have delayed ductal branching but no effect on MMTV-Wnt1-induced mammary tumorigenesis.** (A,B) Morphometric analysis of the number of terminal end buds (A) in glands of virgin 5-week-old females (*n*=5 WT and 8 *Lrp5*^ΔLDLRA/ΔLDLRA^) and number of branches (B) per gland of virgin 12-week-old female glands (*n*=12 WT and 10 *Lrp5*^ΔLDLRA/ΔLDLRA^). MMTV-Wnt1 transgenic female mice that are *Lrp5*^+/+^ (*n*=11) or *Lrp5*^ΔLDLRA/ΔLDLRA^ (*n*=9) were palpated weekly, and dates of tumor appearance were recorded. Graphs show individual values with the median of each group indicated by the middle line; upper and lower lines represent s.e.m. ns, not significant; ***P<0.001 (negative binomial regression). (C) Data are plotted as the proportion of mice in each genotype remaining tumor free as a function of days of age.

LRP5-deficient mice have clear deficits and delays in mammary development (Lindvall et al., 2006). To assess the requirement for the LRP5 class A repeats in this process, we collected mammary glands from virgin juvenile (5-week) and mature (12-week) females. At the juvenile stage, we counted the number of terminal end buds (TEBs), which are important for linear ductal elongation. TEBs are bulbous structures at the distal end of the duct composed of myoepithelial and epithelial cells that interact with the stroma of the fat pad to determine the length and patterning of the mammary gland (Williams and Daniel, 1983). Although there was more variability in the number of TEBs in *Lrp5*^ΔLDLRA/ΔLDLRA^ glands, there was not a statistical difference in the number of TEBs compared to that in WT littermates (Fig. 5A). This is different from our finding in the LRP5 knockout animals, which had a significant reduction in TEBs, suggesting that the type A repeats are not necessary for the normal efficiency of TEB formation.

After puberty, the mature ductal structure becomes more complex and fills the fat pad (Macias and Hinck, 2012). To assess differences in ductal complexity, we counted the number of branches per gland past the lymph node in the 4th mammary gland, which is a commonly used reference point because of its reproducible position (Tolg et al., 2018). *Lrp5*^ΔLDLRA/ΔLDLRA^ glands had a 43% decrease in branching compared to that in the glands of WT littermates (Fig. 5B). This reduction is similar to that seen in LRP5 knockout glands at the same age (Lindvall et al., 2006). Histologically the *Lrp5*^ΔLDLRA/ΔLDLRA^ mammary gland cellular morphology looks like that of controls except for less branching.

### Homozygosity for the *Lrp5*^ΔLDLRA^ allele does not affect MMTV-Wnt1-induced tumorigenesis

We previously evaluated the role of *Lrp5* and *Lrp6* in a mammary tumor model in which the ectopic expression of Wnt1 under the control of the MMTV promoter (MMTV-Wnt1) causes the rapid onset of mammary tumors (Lindvall et al., 2006, 2009; Badders et al., 2009). When we created MMTV-Wnt1; *Lrp5*^ΔLDLRA/ΔLDLRA^ mice, we found that the onset of tumors was not altered relative to that in mice wild type for *Lrp5* (Fig. 5C). This was in sharp contrast to MMTV-Wnt1 transgenic female transgenic mice homozygous for a null allele of *Lrp5*, in which there was a clear delay in tumorigenesis (Lindvall et al., 2006).

### Evaluating *Lrp5*^ΔLDLRA^ signaling mechanisms *in vitro*

**Fig. 6.**
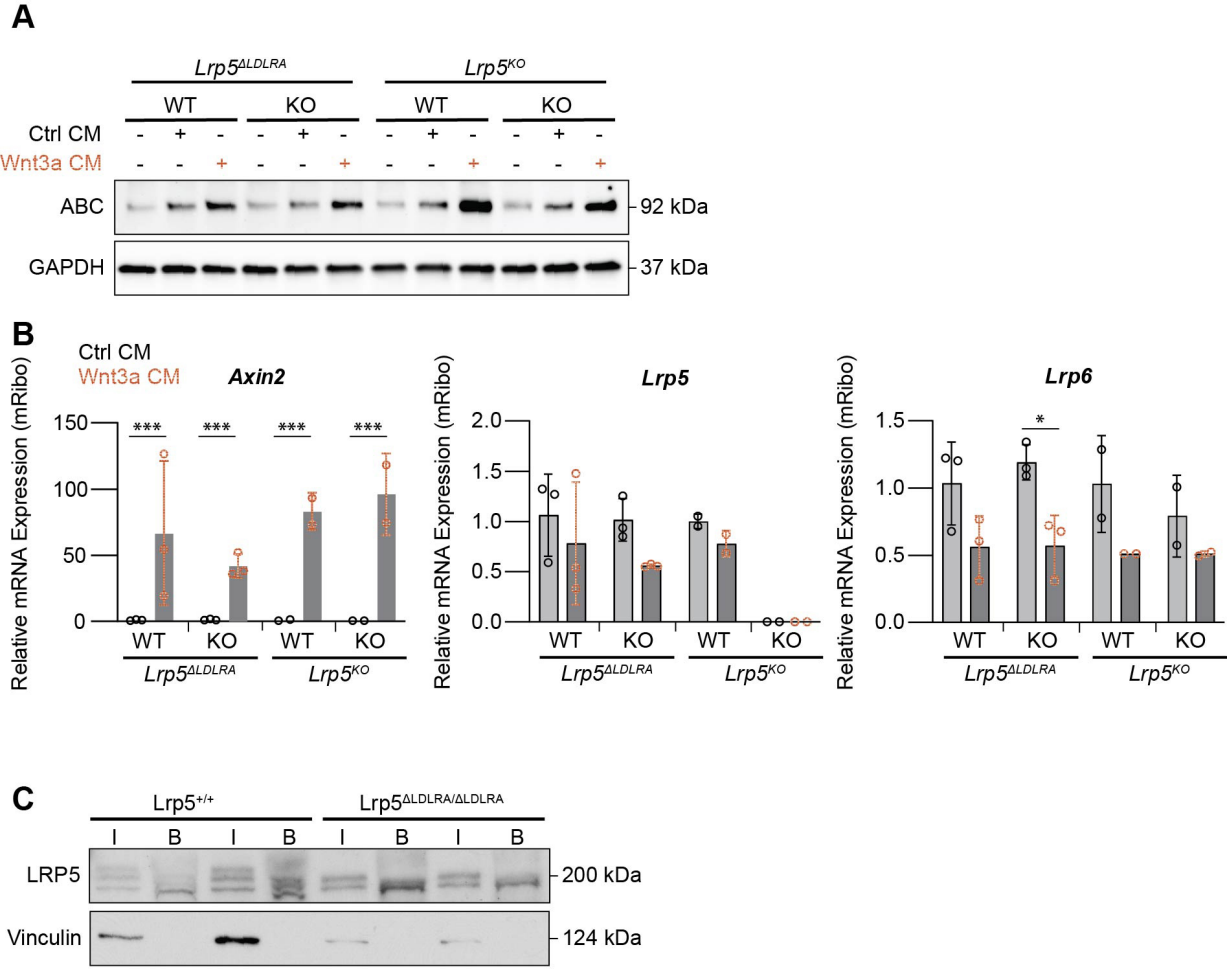
**LRP5 protein is present on the cell membrane and responds to Wnt3a conditioned medium (CM), which induces expression of Wnt/β-catenin targets in *Lrp5*^ΔLDLRA/ΔLDLRA^ MEFs.** (A) *Lrp5*^ΔLDLRA^ and *Lrp5*^KO^ wild-type (WT) and homozygous knockout (KO) MEFs were cultured for 48 h in LGK974 (1 µM) before treatment with control CM, or Wnt3a CM or no treatment for 2 h. Cytosolic fractions were collected to analyze active β-catenin (ABC) with GAPDH used as a loading control. (B) RNA was isolated from the treated MEFs, and *Axin2*, *Lrp5* and *Lrp6* expression levels were quantified relative to those of ribosomal controls (*n*=3 biological *Lrp5*^ΔLDLRA^ and *n*=2 biological *Lrp5*^KO^ replicates/group with three technical replicates each). *P<0.05 and ***P<0.001 (linear mixed effects model). (C) Cell surface proteins from *Lrp5*^+/+^ and *Lrp5*^ΔLDLRA/ΔLDLRA^ MEFs were biotinylated at 4°C for 30 min and lysed, and biotinylated proteins were immunoprecipitated with avidin beads. Bulk (B) and immunoprecipitated (I) proteins were analyzed by western blotting for LRP5, with vinculin as a cytoplasmic control. Two biological replicates per genotype are shown.

To understand how the LDLR type A repeats are involved in Wnt/β-catenin signaling, we generated murine embryonic fibroblasts (MEFs) from both *Lrp5*^ΔLDLRA^ and *Lrp5*^KO^ lines. We wanted to understand whether the modified LRP5 proteins affect the cellular response to Wnt3a conditioned medium (CM). To test this, we inhibited endogenous Wnt ligand production by treating the cells with LGK974 for 48 h before CM treatment (Liegel et al., 2023). After the cells were either left untreated or treated with control or Wnt3a CM for 2 h, they were processed for analysis of cytoplasmic proteins. The data show that, regardless of *Lrp5* genotype, cytosolic β-catenin levels are like those in WT littermate control cells (Fig. 6A). The stabilization of β-catenin is likely due to functional LRP6 protein sustaining the Wnt/β-catenin signal in these cells, as cells lacking both *Lrp5* and *Lrp6* do not stabilize β-catenin in response to Wnt3a (Chen and He, 2019). We also examined *Axin2*, *Lrp5* and *Lrp6* expression levels in these same cells. Wnt3a CM treatment in all cell populations increased relative *Axin2* levels significantly, with little effect on *Lrp5* or *Lrp6* expression (Fig. 6B).

To confirm LRP5 localization to the cell membrane, we performed surface biotinylation of *Lrp5*^ΔLDLRA^ and *Lrp5^+/+^* MEFs and bone marrow stromal cells (BMSCs) under cold conditions to prevent internalization of membrane-associated proteins. BMSCs were included because osteoblasts, the cell type in which *Lrp5* and *Lrp6* regulate bone mass, are derived from this lineage. Following cell lysis, biotinylated surface proteins were isolated using avidin-agarose beads and analyzed by immunoblotting for LRP5 in input and pulldown fractions. LRP5 protein was detected at the membrane at similar levels in *Lrp5*^ΔLDLRA^ and *Lrp5^+/+^* cells (Fig. 6C; Fig. S4). These results demonstrate that deletion of the LDLR type A repeats does not impair LRP5 trafficking to the plasma membrane.

We further evaluated the signaling activity of *LRP5*^ΔLDLRA^ in HEK293T cells lacking both endogenous LRP5 and LRP6 (HEK293T^LRP5/6-less^). This system enabled us to assess signaling in the complete absence of endogenous LRP5 or LRP6. We generated cDNA expression plasmids encoding either *LRP5*^ΔLDLRA^ or wild-type LRP5 and transfected them into HEK293T^LRP5/6-less^ cells. Despite comparable protein expression levels from these constructs, *Lrp5*^ΔLDLRA^ signaling activity was diminished by 45-65% relative to that in wild type in multiple contexts (Fig. S5A,B). These include co-transfection with plasmids expressing previously characterized fusion proteins used to study LRP5/6 receptor signaling, such as Wnt8-Fzd5 and Wnt3a-Fzd5 fusions (Holmen et al., 2005, 2002). These findings indicate that *LRP5*^ΔLDLRA^ retains partial signaling activity and, in some contexts, is not a complete loss-of-function (null) allele. We speculate that protein overexpression associated with transient transfection in HEK293T cells is sufficient to mask the loss of function observed in primary cells and in several tissues of mice homozygous for the *Lrp5*^ΔLDLRA^ allele.

## DISCUSSION

LRP5 was first identified in the late 1990s based on its homology to LDLR, and thus several initial studies focused on its role in lipoprotein and glucose metabolism (Dong et al., 1998; Hey et al., 1998; Kim et al., 1998; Magoori et al., 2003; Fujino et al., 2003). In 2000, three reports independently established roles for LRP5, the highly homologous LRP6 protein, and their *Drosophila* homolog, *Arrow*, in Wnt signaling. However, these reports did not evaluate LDLR type A repeat function (Wehrli et al., 2000; Tamai et al., 2000; Pinson et al., 2000). A requirement for the LRP6 LDLR type A repeats to increase β-catenin levels was specifically examined after transient transfection of a plasmid expressing a mutant lacking these repeats in HEK293T cells (Brennan et al., 2004). In this setting, the absence of type A repeats did not diminish the ability of LRP6 to increase β-catenin levels in cells. However, it is important to note that transient transfection of HEK293T cells typically results in expression of supraphysiological amounts of protein from the transfected plasmid.

We have addressed the role(s) of the LRP5 type A repeats in the context of endogenous expression by creating and characterizing mice specifically and uniquely lacking the type A repeats. In several settings, the type A repeats are necessary for normal LRP5 function. These include the establishment of normal bone mineral density and the development of the retinal vascularization. Defects in these processes are the chief phenotypic manifestations associated with OPPG, and the *Lrp5*^ΔLDLRA/ΔLDLRA^ mice model aspects of OPPG similarly to mice homozygous for the null allele of *Lrp5*. In addition, the genetic interaction between null alleles of *Lrp5* and *Lrp6*, which results in early embryonic lethality, is also observed in embryos homozygous for the null alleles of *Lrp6* and *Lrp5*^ΔLDLRA^.

The requirement for the type A repeats for LRP5 functions in these settings contrasts with what is seen in MMTV-Wnt1-induced mammary tumorigenesis and limb development during late embryogenesis. We previously found that homozygosity for a null allele of *Lrp5* inhibits tumor progression in the MMTV-Wnt1 mouse mammary tumor model (Lindvall et al., 2006). In contrast, homozygosity for the *Lrp5*^ΔLDLRA^ allele had no demonstrable effect on tumor latency, with *Lrp5*^ΔLDLRA/ΔLDLRA^ mice developing tumors at a pace indistinguishable from that in their WT littermates. One explanation for this could be that the ectopic expression of Wnt1 from the strong MMTV LTR promoter is associated with supraphysiological levels of Wnt1 protein. These ectopic expression levels could reduce the necessity for the type A repeats. For example, Wnt ligands associate with lipoproteins to facilitate trafficking through the extracellular space (Kaiser et al., 2019; Neumann et al., 2009; Panakova et al., 2005; Mehta et al., 2021). Perhaps the LRP5 type A repeats, which bind apolipoproteins (Kim et al., 1998; Joiner et al., 2013), contribute to the association between Wnt and LRP5 but are not absolutely required for the interaction. Thus, under very high levels of Wnt expression, they are not necessary for functional activity.

Although this study establishes the requirement for the LRP5 LDLR type A repeats in development, the fact that the highly related LRP6 protein contains similar LDLR type A repeats and functions as a Wnt co-receptor may mean that a fuller assessment of the roles of these type A repeats in biology will require the creation of *Lrp6*^ΔLDLRA^ mice. We have noted that the same strategy used to create the *Lrp5* allele uniquely and specifically lacking the type A repeat can also be applied to create a similar allele of *Lrp6*. Future studies could focus on creating and characterizing such *Lrp6* mice and evaluating mice homozygous for alleles of *Lrp5* and *Lrp6* lacking the type A repeats. We have identified redundancy in LRP5 and LRP6 function in numerous contexts, including early osteochondral differentiation (Joeng et al., 2011), the establishment of liver zonation (Yang et al., 2014) and intestinal epithelial differentiation (Zhong et al., 2012). Evaluating the requirements for the LRP5 and LRP6 type A repeats in these settings could provide additional insights into Wnt receptor signaling and further inform approaches to therapeutically target these receptors (Post et al., 2024; Zhong et al., 2021).

## MATERIALS AND METHODS

### Animals

CRISPR gene editing generated animal models lacking the LDLR type A (*Lrp5*^ΔLDLRA^) repeats within *Lrp5*. This region is encoded in exons 18 and 19 exclusively. Two different founder lines, *Lrp5*^em4Vari^ and *Lrp5*^em5Vari^, were generated by deleting exons 18 and 19 of *Lrp5* using a modified CRISPR/Cas9 protocol (Cong and Zhang, 2015). Briefly, we designed two sgRNAs targeting intron 17 of *Lrp5* (GTGACTTCCCCCGTATACTT) and intron 19 of *Lrp5* (CCAGTCTACAGGGGTCTATT) using MIT's guide sequence generator (crispr.mit.edu). The guide sequence was then cloned into vector pX330-U6-Chimeric_BB-CBh-hSpCas9 (Addgene plasmid #42230; RRID:Addgene_42230; deposited by Feng Zhang). The T7 promoter was added to the sgRNA template prior to synthesis by IDT. The PCR-amplified T7-sgRNA product was used as template for *in vitro* transcription using a MEGAshortscript T7 kit (Thermo Fisher Scientific). The injection mix consisted of Cas9 mRNA (Sigma-Aldrich) (final concentration of 40 ng/μl) and sgRNAs (20 ng/μl each) in injection buffer (10 mM Tris-HCl, 0.1 mM EDTA pH 7.5) injected into the pronucleus of C57BL/6;C3H zygotes. After identifying founders, we backcrossed the line to C57BL/6 twice before intercrossing to generate animals for our study. *Lrp5*^ΔLDLRA^ mice were maintained following institutional animal care and use guidelines, and experimental protocols were approved by the Institutional Animal Care and Use Committee of the Van Andel Institute.

For genotyping the *Lrp5*^ΔLDLRA^ and *Lrp5*^+/+^ alleles, the following primers were used: WT product (484 bp): Lrp5-I17-Fwd (5′-CCAGAAACAGGCAAGGAAGA-3′), Lrp5-E18/19WT-Rev (5′-GCTTCAGACCACAAGTCTGTTTA-3′); knockout product (725 bp): Lrp5-I17-Fwd (5′-CCAGAAACAGGCAAGGAAGA-3′), Lrp5-E20-Rev (5′-AGAGGGTGGCTTGTTGATTT-3′).

For qRT-PCR analysis of founder tissue, the following primers were used: mLRP5_E17-Fwd (5′-GCTGCTCCCACATCTGTATC-3′), mLRP5_E20-Rev (5′-ACGCTGGCAGACAAAGTAG-3′); mGAPDH-Fwd (5′-AGGTCGGTGTGAACGGATTTG-3′), mGAPDH-Rev (5′-GGGGTCGTTGATGGCAACA-3′).

### DXA

We performed whole-body DXA to measure aBMD (in g/cm^2^) and bone mineral content (BMC; in g) for the postcranial skeleton. A PIXImus II bone densitometer (GE Lunar) was used for analysis (Holmen et al., 2004). aBMD values were collected on the same animals at 3 and 6 months of age.

### µCT

For skeletal analysis, femurs were isolated from 3-month-old female and male mice, fixed in 10% neutral-buffered formalin at room temperature for 48 h, and then changed to 70% ethanol before analysis.

The *Lrp5*^ΔLDLR^ models were analyzed using a SkyScan 1172 µCT system (Bruker MicroCT, Kontich, Belgium) (Foxa et al., 2021). Femora were scanned in 70% ethanol using an X-ray voltage of 60 kV, current of 167 µA, and 0.5 mm aluminum filter with a voxel size of 7.98 µm. Femoral images were reconstructed using NRecon 1.7.4.6 (Bruker MicroCT). The mineralized tissue was oriented, and a volume of interest was defined using DataViewer 1.5.6.3 (Bruker MicroCT). Regions of interest (ROIs) were defined for cortical and trabecular bone using CTAn 1.18.8.0 (Bruker MicroCT). A trabecular ROI was drawn in the distal epiphysis for each femur, beginning 0.25 mm proximal to the growth plate and 2.5 mm in height. To define each cortical ROI's position, 45% of the femur length was calculated and used to set the distal end of the region. The ROI was 0.8 mm in height toward the proximal end of the bone, within the midshaft. Trabecular 3D analysis was performed to quantify BMD, bone volume/tissue volume, bone surface/bone volume, trabecular thickness, trabecular separation and trabecular number. Cortical 2D analysis was performed to quantify tissue mineral density, tissue area, bone area, CAF (bone area/tissue area), cross-sectional thickness and bone perimeter.

### Skeletal preparations

Embryos (E18.5) were stained with Alcian Blue and Alizarin Red according to established protocols (Joeng et al., 2011). Briefly, embryos were fixed overnight at 4°C in 70% ethanol, then switched to 95% ethanol for 1 h, followed by acetone overnight at room temperature. Embryos were then stained with Alcian Blue [0.03% (w/v), 80% ethanol, 20% (glacial) acetic acid) for 4 h followed by Alizarin Red (0.005% (w/v) in 1% (w/v) KOH] staining for 4 h. Embryos were then cleared in 1% KOH overnight and transferred to a 50% glycerol: 50% (1%) KOH solution at room temperature until tissue appeared transparent. Stained embryos were imaged using a stereoscope (Nikon, SMX1000) and assessed for gross morphology differences.

### Histology and staining of the vasculature in retinal flat mounts and frozen sections

The retinal vasculature in flat mounts and frozen sections of retinas of 3-month-old WT, heterozygous and homozygous Lrp5^ΔLDLRA^ mice was stained with isolectin B_4_ by the same methods used in our previous studies of *Lrp5* knockout rats (Ubels et al., 2022, 2020). Paraffin sections were stained with Hematoxylin and Eosin (H&E).

### ERG

Electroretinograms were recorded using the Diagnosys Celeris System (Diagnosys, Lowell, MA, USA). Mice were dark adapted overnight prior to testing. Pupil dilation was achieved with topical 1% tropicamide, and corneal anesthesia was induced with 0.5% proparacaine. Anesthesia was administered via intraperitoneal injection of ketamine (90 mg/kg) and xylazine (10 mg/kg). A drop of 0.3% hypromellose (GenTeal Tears, Alcon, Fort Worth, TX, USA) was applied to each cornea to maintain hydration and facilitate contact with the electrode stimulators.

Scotopic ERG responses were recorded using stimulus intensities of 0.01, 0.1, 1, 10 and 32 cd·s/m². Body temperature was maintained at 37°C throughout testing using the built-in heating platform of the Celeris System.

### Mammary whole mounts

Inguinal mammary glands from 12-week-old and 5-week-old females were isolated to examine ductal branching and the number of terminal end buds, respectively (Lindvall et al., 2006). Briefly, mammary tissue was spread on a glass slide and fixed overnight in Carnoy's fixative (60% ethanol, 30% chloroform, 10% glacial acetic acid). The tissue was washed in 70% ethanol for 15 min, hydrated, stained with carmine alum (24 h), dehydrated and cleared in xylene.

### Antibodies

Antibodies against LRP5 D80F2 (Cell Signaling Technology, 5731, RRID:AB_10705602), LRP6 C47E12 (Cell Signaling Technology, 3395, RRID:AB_1950408), non-phospho (active) β-catenin (Ser33/37/Thr41) D13A1 (Cell Signaling Technology, 8814, RRID:AB_11127203), β-actin-HRP13E5 (Cell Signaling Technology, 5125, RRID:AB_3099713), GAPDH 14C10 (Cell Signaling Technology, 2118, RRID:AB_561053) and vinculin (Cell Signaling Technology, 4650, RRID:AB_10559207) were used at a dilution of 1:1000.

### Plasmid generation and validation

The C-terminal V5-tagged hLRP5-WT and pCS2+DNXWnt8-hFzd5 constructs were previously generated (Holmen et al., 2002). To generate pCS2+mWnt3a-hFzd5, we replaced the DNXWnt8 with mWnt3a.

To generate the C-terminal V5-tagged hLRP5-ΔLDLR plasmid, site-directed mutagenesis was performed on the WT plasmid using a Q5 Site-Directed Mutagenesis Kit (NEB, E0554) with the following primers: LRP5-ΔLDLR-F (5′-ATCACCAAGCCGCCCTCA-3′) and LRP5-ΔLDLR-R (5′-TCCACAGGTCAGCAGGTTC-3′).

Clones were verified by whole-plasmid sequencing (Plasmidsaurus), and expression of the constructs was confirmed by western blotting.

### Cell lines and primary cells

Immortal HEK293 STF cells (ATCC, CRL-3249), HEK293T BR *LRP5/6* null cells (Chen and He, 2019) (a gift from Xi He, Boston Children's Hospital, Boston, MA, USA), L cells stably expressing Wnt3a (ATCC, CRL-2647), untransfected L cells (ATCC, CRL-2648) (Willert et al., 2003) and primary MEFs (Durkin et al., 2013) were cultured in Dulbecco's modified Eagle's medium (Thermo Fisher Scientific) supplemented with 10% (v/v) fetal bovine serum (FBS; Corning), 100 units/ml penicillin (Thermo Fisher Scientific) and 100 μg/ml streptomycin (Thermo Fisher Scientific).

BMSCs were harvested from the long bones of 11-week-old animals and cultured in α-MEM supplemented with 20% FBS and 1% penicillin/streptomycin (Michalski et al., 2016).

All cells were maintained in a humidified incubator at 37°C with 5% CO₂.

### CM preparation

Wnt3a and CM were prepared using L cells stably transfected with a Wnt3a-expressing plasmid or untransfected L cells. Briefly, L cells were grown in media containing 10% FBS for 4 days. CMs were then collected, centrifuged to remove cellular debris, filtered and stored at 4°C for later use.

### Cell lysis for protein expression analysis

For protein expression analysis, detergent-solubilized cell lysates were prepared by resuspending cells in PPHB lysis buffer [50 mM Na₂HPO₄, 1 mM sodium pyrophosphate, 20 mM NaF, 2 mM EDTA, 2 mM EGTA, 1% Triton X-100, 1 mM DTT and protease inhibitors (Roche, 11836170001)], followed by centrifugation (Liu et al., 2009).

### Analysis of cytosolic β-catenin activity

For analysis of cytosolic β-catenin activity, MEF cells were pretreated with 1 μM porcupine inhibitor LGK974 (MedChemExpress, HY-17545) for 48 h before plating in six-well tissue culture plates. After plating, all cells continued to receive LGK974 treatment, with separate groups additionally treated for 2 h with either Wnt3a CM, control CM or no CM treatment.

Cells were lysed in isotonic lysis buffer (10 mM Tris-HCl pH 7.4, 140 mM NaCl, 2 mM DTT and protease inhibitors), and cytosolic extracts were isolated and subjected to immunoblotting for active β-catenin as previously described (Shimizu et al., 1997).

### Cell surface protein biotinylation

For biotinylation of cell surface proteins, BMSCs or MEFs were plated in 10 cm dishes. All cell labeling, protein extraction and precipitation steps were performed in a cold room at 4°C.

Once the cells reached ∼70% confluency, they were rinsed three times with ice-cold PBS and incubated with 2 ml of 1 mg/ml EZ-Link™ Sulfo-NHS-LC-Biotin (Thermo Fisher Scientific, 21335) for 30 min with gentle rocking. After incubation, cells were washed three times with PBS containing 100 mM glycine to quench unreacted biotin.

Proteins were then lysed in PPHB lysis buffer containing protease inhibitors. A portion of the lysate was retained as input control, while the remaining lysate containing biotinylated proteins was incubated with NeutrAvidin agarose beads (Thermo Fisher Scientific, 29200) overnight at 4°C. Precipitates were washed three times with lysis buffer and used for subsequent immunoblotting analysis.

### Luciferase reporter assay

To perform the luciferase reporter assay, HEK293 STF cells and HEK293T BR *LRP5/6* null cells were plated in 12-well plates and transfected in suspension using XtremeGene HP (Roche) with a total of 300 ng DNA per well. Each transfection contained 100 ng of the indicated plasmid(s), 100 ng pCDNA3.1-lacZ and sufficient empty vector DNA to normalize the total amount of DNA across all conditions.

After 24 h, cell lysates were harvested and analyzed using the Luciferase Assay System (Promega). Luciferase activity was measured with a Synergy Neo HTS multimode microplate reader (BioTek) and normalized for transfection efficiency by measuring β-galactosidase activity, as previously described (Chen and He, 2019). Both luciferase and β-galactosidase activities were measured in triplicate, and transfections were also performed in triplicate.

### qRT-PCR

qRT-PCR was performed using standard methods. Total RNA was extracted from MEFs using a Quick-RNA MiniPrep Plus Kit (Zymo, R1057), and reverse transcription was carried out using a High-Capacity cDNA Reverse Transcription Kit (Thermo Fisher Scientific, 4368814).

Real-time PCR amplification was performed with SYBR Select Master Mix (Thermo Fisher Scientific, 4472908) on a QuantStudio™ 6 Pro Real-Time PCR System (Thermo Fisher Scientific).

Primers for mouse *Axin2*, *Lrp5* and *Lrp6* were selected using PrimerBank and were as follows: *Axin2* Forward, 5′-TCATTTTCCGAGAACCCACCGC-3′; *Axin2* Reverse, 5′-GCTCCAGTTTCAGTTTCTCCAGCC-3′; *Lrp5* Forward, 5′-AATCGAAAGCTGTGACCTCTC-3′; *Lrp5* Reverse, 5′-CCTACTGGCTGTACGATGTTG-3′; *Lrp6* Forward, 5′-CCATTTGTGTTTGATGTCTCCAG-3′; *Lrp6* Reverse, 5′-AATTCGCCTCAAGTCTGTCC-3′.

### Statistical analyses

DEXA, µCT and mammary branching data were analyzed using R v 4.4.0. All models were stratified by sex, and the R package emmeans (Lenth, 2024) was used to test individual hypotheses. For the µCT analysis, methods were based on the scale of the outcome; percentage data such as CAF were analyzed via beta regression using the betareg package (Cribari-Neto and Zeileis, 2010), and the other outcomes were analyzed via robust linear regression using the MASS package (Venables and Ripley, 2002) with natural log (y +0.01) transformations to improve the normality of the residuals. All µCT outcomes were then Benjamini–Hochberg false discovery rate (FDR) adjusted to account for multiple testing and maintain a 5% false discovery rate. DEXA data were analyzed using linear mixed-effects models with random intercepts for each animal via the lme4 package (Bates et al., 2015); random slopes were also included for longitudinal data with more than two timepoints. To test whether the number of terminal end branches at 5 weeks and/or the number of mammary branches at 12 weeks differed between groups, negative binomial regression via the MASS package was used.

Quantitative PCR data were analyzed using a linear mixed-effects model via the R package lme4. Each gene (*Axin2*, *Lrp5* and *Lrp6*) was first normalized to mouse 18S ribosomal RNA (mRibo), which served as the internal reference gene. For each gene, the cycle threshold (CT) value was normalized to that of mRibo by calculating ΔCT=CT(target)–CT(mRibo). A random intercept for each founder was included to account for relatedness among the animals.

For the ERG analyses, tests of statistical significance between groups were performed using Prism software (GraphPad Software). Significances of differences between groups (*P*≤0.05 was considered significant) were determined using two-way ANOVA with Sidak correction.

### Use of AI tools

Grammarly and ChatGPT were used to edit the text and improve clarity.

## Supplementary Material

10.1242/dmm.052280_sup1 Supplementary information
