# Supplementary material for: The class A repeats of LRP5 are required for normal development of bone, retinal vasculature and mammary gland in vivo
Source: Dis Model Mech. 2025 Nov 11;18(11):dmm052280. doi: 10.1242/dmm.052280 (PMC12661645; doi:10.1242/dmm.052280)
Supplement: Supplementary information [file dmm-18-052280-s1.pdf]

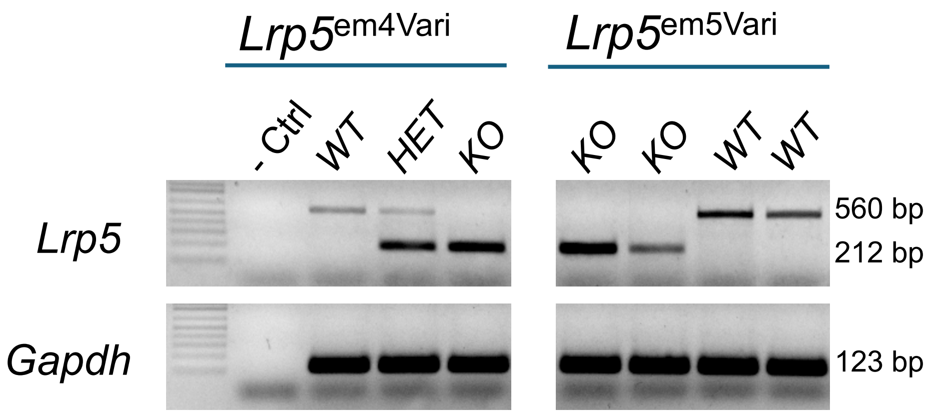

Fig. S1. RNA expression of *Lrp5* in *Lrp5*$^{\Delta LDLRA}$ MEFs.

PCR using primers in Lrp5 exons 17 and 20 produced a 560 bp fragment for wild-type (WT) and a 212 bp fragment for the Lrp5$^{\Delta LDLRA}$ allele. Samples include WT, heterozygous (Lrp5$^{\Delta LDLRA/+}$; Het), and homozygous knockout (Lrp5$^{\Delta LDLRA/\Delta LDLRA}$; KO) cells. Gapdh amplification served as a loading control.

Disease Models & Mechanisms • Supplementary information

A Early embryonic lethality

Time point	# of litters	$Lrp5^{\Delta LDLRA/\Delta LDLRA}$ $Lrp6^{+/+}$	$Lrp5^{\Delta LDLRA/\Delta LDLRA}$ $Lrp6^{KO/+}$	$Lrp5^{\Delta LDLRA/\Delta LDLRA}$ $Lrp6^{KO/KO}$	Unknown genotype
E7.5	1	1	3	4*	
E9.5	2	5	5	2*	3**
E11.5	2	3	4		8**

* Resorbing or malformed
** No embryo to genotype (empty decidua)

B

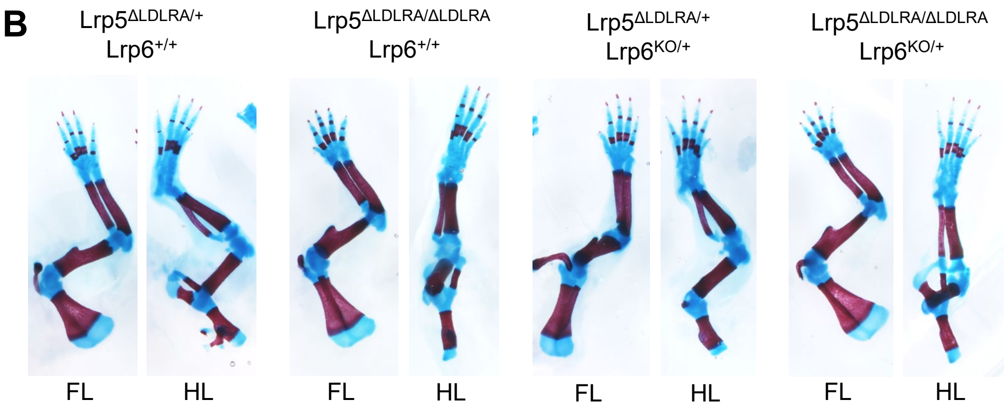

Fig. S2. *$Lrp5^{\Delta LDLRA}$ $Lrp6^{KO}$* compound knockout animals are not exactly like $Lrp5^{KO}$ $Lrp6^{KO}$ animals. (A)$Lrp5^{\Delta LDLRA/\Delta LDLRA}$ $Lrp6^{KO/KO}$ embryos are embryonic lethal. Embryos were collected at E7.5, E9.5, and E11.5 and genotyped. (B)$Lrp5^{\Delta LDLRA/\Delta LDLRA}$ $Lrp6^{KO/+}$ animals have normal limb patterning at 3 months of age (n=13). Forelimbs (FL) and hindlimbs (HL) were collected and stained with alizarin red and alcian blue.

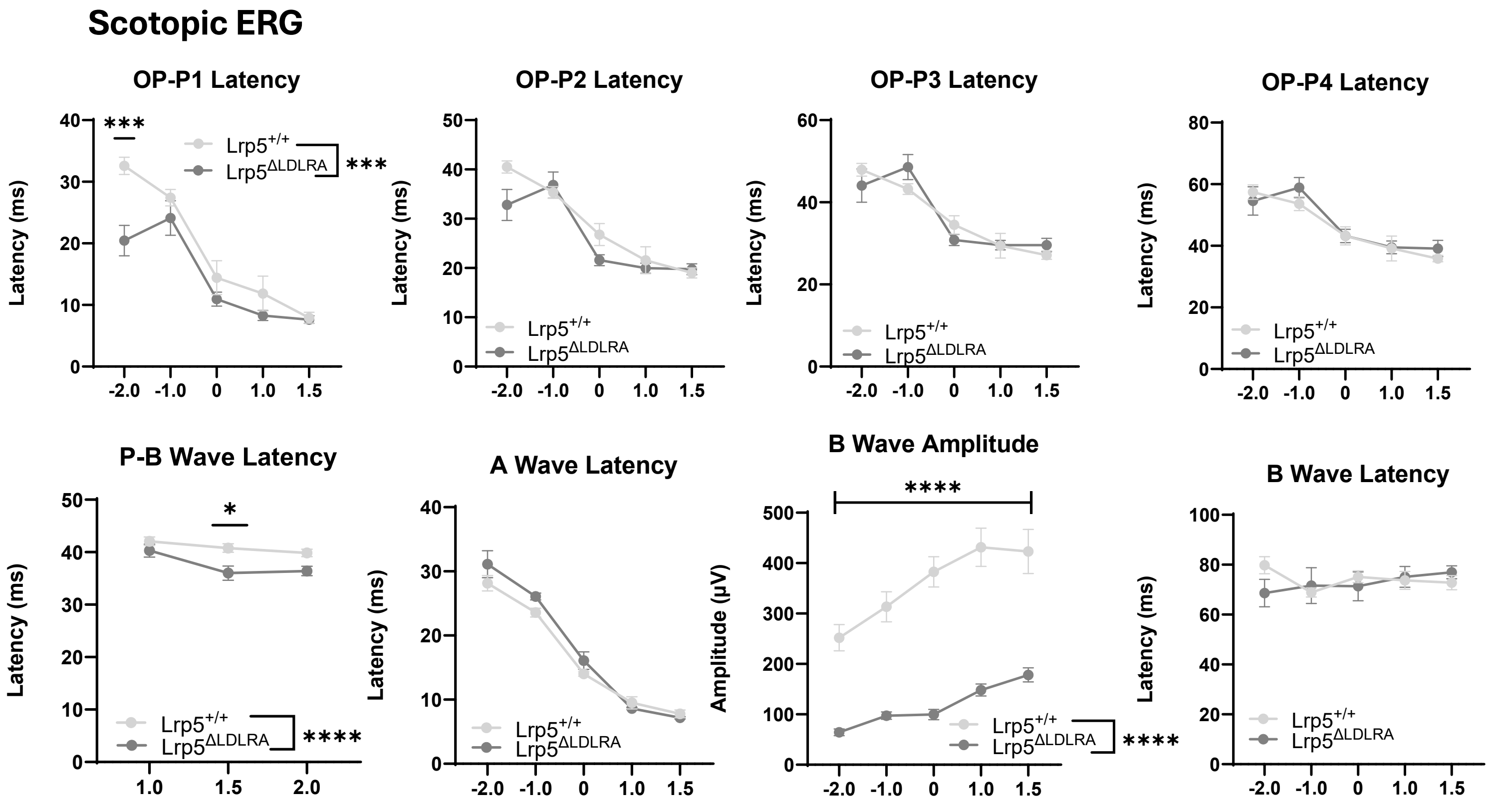

Fig. S3. ERG assessment of retinal function under scotopic conditions. Scotopic ERG responses, representing rod-mediated visual function, were assessed by measuring a-wave and b-wave amplitudes and latencies. The representative traces and quantifications indicate differences in retinal response under low-light conditions between WT and Lrp5ΔLDLRA/ΔLDLRA animals.

Data are presented as mean ± SEM. Statistical significance was determined using a two-tailed Student's t test for comparisons between WT and knockout groups at each intensity. For multiple comparisons, one-way ANOVA with Sidak correction was used. Significance levels: $P \leq 0.05$ (*), $P \leq 0.01$ (**), $P \leq 0.001$ (***), and $P \leq 0.0001$ (****).

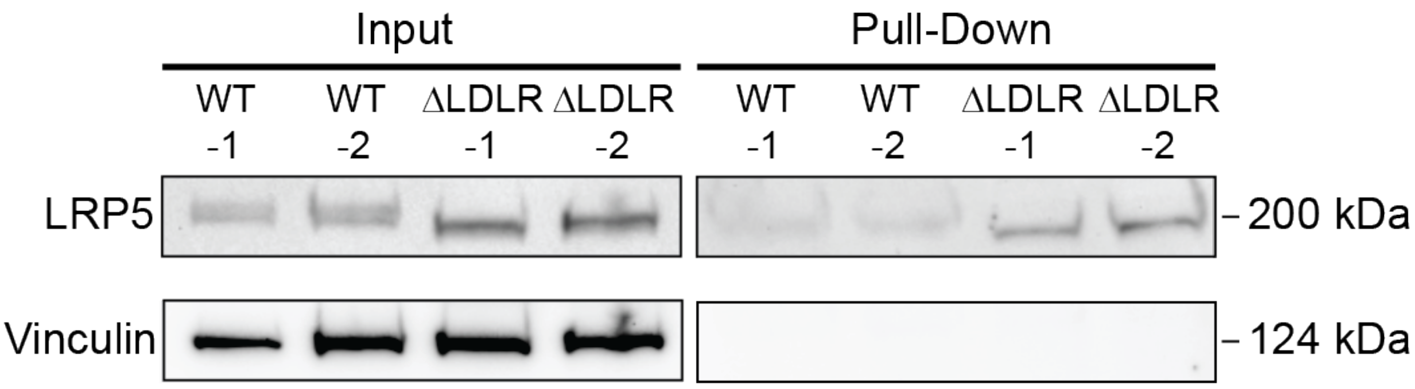

Fig. S4. Membrane Expression of *Lrp5* in wild-type and *Lrp5*$^{\Delta LDLRA}$ BMSCs. *Lrp5* wild-type (WT) and *Lrp5*$^{\Delta LDLRA/\Delta LDLRA}$ BMSCs were surface-biotinylated at 4°C for 30 minutes, lysed, and biotinylated proteins were immunoprecipitated with avidin beads. Input and pull-down samples were analyzed by western blot for LRP5, with Vinculin as a cytoplasmic control. Two biological replicates per genotype are shown.

Disease Models & Mechanisms • Supplementary information

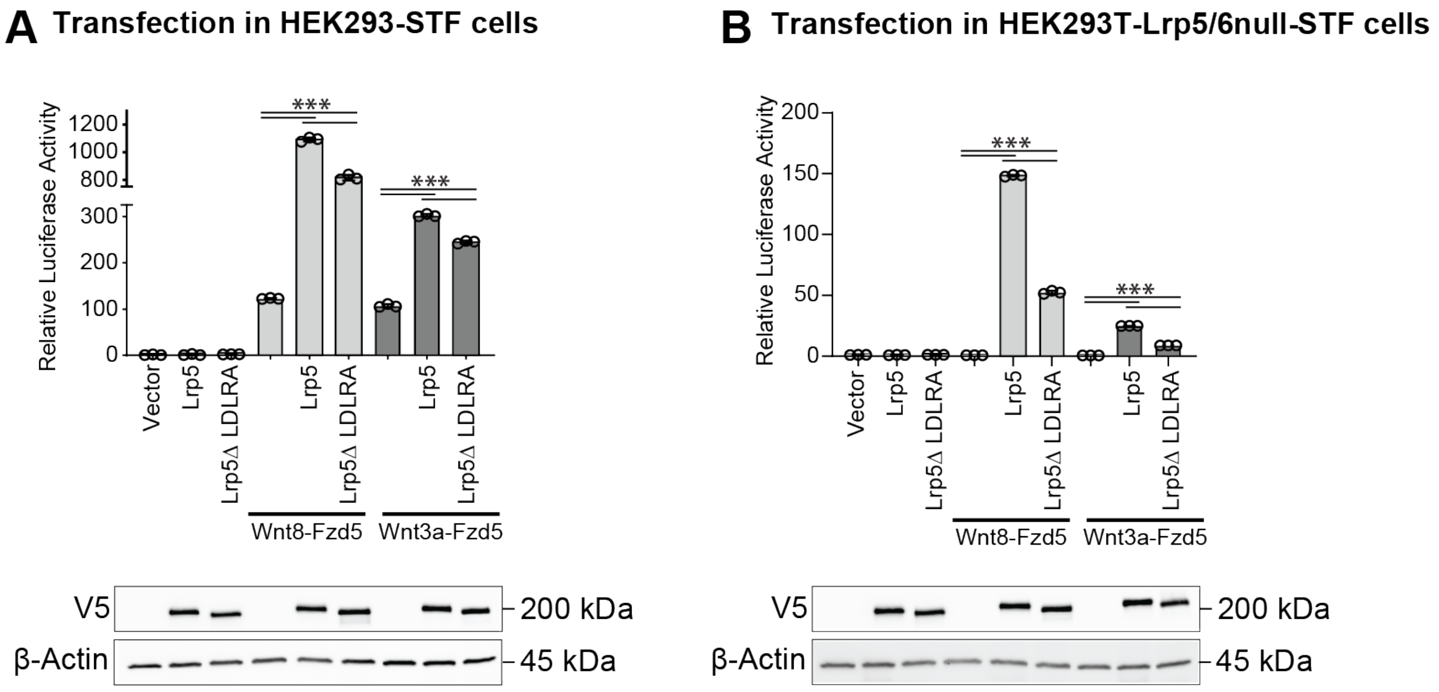

Fig. S5. *Lrp5*$^{\Delta LDLRA}$ acts like a hypomorph in HEK293 reporter cell models. *Lrp5* wild-type (WT) *and Lrp5*$^{\Delta LDLRA}$ V5-tagged plasmids were co-transfected with Wnt8-Fzd5 or Wnt3a-Fzd5 fusion plasmids in HEK293-STF(A) and HEK293T-Lrp5/6null-STF(B) cells. Luciferase activity was normalized to empty vector. Data are mean ± SD of three technical replicates. Two-tailed Student's t-test: ***$p < 0.001$.

Disease Models & Mechanisms • Supplementary information
